# Draft genome sequences of three emerging beta-lactamase-producing *Escherichia coli* in the camel production system in Northern Kenya

**DOI:** 10.12688/f1000research.127990.1

**Published:** 2022-11-30

**Authors:** Rachael Gachogo, Irene Karegi, Brian Ogoti, Victor Musyoki, Dino Martins, Frank Onyambu, Joseph Kamau

**Affiliations:** 1 Center for Molecular Biosciences and Genomics, Nairobi, Kenya; 2 Division of immunology, Department of Human Pathology, University of Cape Town, Cape Town, South Africa; 3 One Health Center, Institute of Primate Research, Nairobi, Kenya; 4 Department of Biochemistry, Jomo Kenyatta University of Agriculture and Technology, Juja, Kenya; 5 Center for Microbiology, Washington State University, Nairobi, Kenya; 6 Department of Medical Microbiology, University of Nairobi, Nairobi, Kenya; 7 Mpala Research Center, Nanyuki, Kenya; 8 Meru University of Science and Technology, Meru, Kenya

**Keywords:** E.coli, beta-lactamase, AMR, genome, whole genome sequencing, camel

## Abstract

We report the draft genome sequences and annotation of three beta-lactamase-producing *Escherichia coli* ( *E.coli*) strains isolated from fecal samples of healthy camels in Laikipia county, Kenya. This data adds to the online genome resources to support the ongoing antimicrobial resistance surveillance in the livestock-wildlife interface.

## Introduction

Antimicrobial resistance (AMR), especially on the readily available beta-lactam antibiotics continues to threaten effective healthcare management and global economic success in livestock farming ( Fashae et al., 2021; Kiiru et al., 2012). Recently, concerted efforts have been employed to explore the AMR situation following a One Health Approach. However, there still exists a gap in whole genome sequencing data on *Escherichia coli* ( *E.coli*) harboring AMR genes in camels ( *Camelus dromedaries*), a key component of livestock farming in arid and semi-arid regions of Northern Kenya.

## Methods

Here, we report three draft genome sequences of beta-lactamase-producing *E.coli* isolated from camel fecal samples in Laikipia county, Northern Kenya. Fecal samples were collected from healthy camels reared under both ranching systems and pastoralism, transported in Cary Blair media (HiMedia Lab, Mumbai, India) and stored at 4 ^o^C awaiting processing.

The samples were cultured on MacConkey agar plate (HiMedia Lab, Mumbai, India) and incubated at 37°C for 18 hours. A single colony per MacConkey agar plate that displayed *E. coli* traits morphologically was identified through Gram staining and the IMViC (indol, methyl red, Voges Proskauer, and citrate) biochemical method and subjected to antimicrobial susceptibility testing (AST) against the beta-lactam antibiotic spectrum. Briefly, antimicrobial susceptibility testing (AST) was performed on Mueller Hinton agar (HiMedia Lab, Mumbai, India) using Kirby Bauer disk diffusion method. Antibiotics tested included, Ampicillin-10 μg (AMP), Chloramphenicol-30 μg (CHL), Tetracycline-30 μg (TCY), Gentamycin-10 μg (GEN), Streptomycin-10 μg (STR1), Trimethoprim-sulfamethoxazole-25 μg (SXT), Norfloxacin-10 μg (NOR), Ciprofloxacin-5 μg (CIP), Cefaclor-30 μg (CEC), Ceftriaxone-30 μg (CRO), Cefotaxime-30 μg (CTX), Cefuroxime-30 μg (CMX), Cefepime-30 μg (FEP), Amoxicillin–Clavulanate-20/10 μg (AMC), and Ceftazidime-30 μg (CA).

Production of beta-lactamase genes was assessed by conducting PCR to confirm genes encoding for CTX-M, TEM, CMY, SHV and OXA for the resistant isolates ( Livermore et al., 2007). Briefly, a 20ul PCR reaction was prepared by adding 10ul of the Platinum II Hot-Start PCR Master Mix (Invitrogen, Carlsbad, CA, U.S), 0.4 ul of 10nmol of forward and reverse primers, 4ul of Platinum GC enhancer, 0.2ul nuclease free water and 5ul DNA. Cycling was performed on Veriti™ Dx 96-well Thermal Cycler (Thermofisher, Waltham, Massachusetts, U.S) using primers and PCR conditions as in Table 1. The respective gene fragment sizes were confirmed in 1% agarose gel (Invitrogen, Carlsbad, CA, U.S)

**Table 1.** Primers used for detection of blaTEM, blaCTX-M, blaCMY, blaOXA, and blaSHV gene(s) minutes (min), Seconds (sec), base pairs (bp).

Primers	Oligonucleotide Sequence (5′-3′)	Annealing Temperature	PCR conditions	Product Size (bp)
TEM	F-ATGAGTATTCAACATTTCCG R-CTGACAGTTACCAATGCTTA	55 °C	94 ^o^C: 2 min 94 ^o^C: 15 Sec X35 Annealing Temperature: 15 Sec X35 68 ^o^C:15 Sec X35 4 ^o^C:10 min	840
SHV	F-GGTTATGCGTTATATTCGCC R-TTAGCGTTGCCAGTGCTC	50 °C		854
CTX-M	F-ATGTGCAGYACCAGTAARGT R-TGGGTRAARTARGTSACCAGA	60 °C		593
CMY	F-ATGATGAAAAAATCGTTATGC R-TTGCAGCTTTTCAAGAATGCGC	55 °C		1200
OXA	F-TCAACTTTCAAGATCGCA R-GTGTGTTTAGAATGGTGA	62 °C		820

Genomic DNA of beta-lactamase producing *E.coli* was extracted using Isolate II Genomic DNA Kit (Bioline) and sequencing performed using Oxford Nanopore Technologies (ONT). Briefly, manufacturer’s sample barcodes (Native Barcoding Expansion 1-12) (EXP-NBD104) and sequencing adapters (Ligation Sequencing kit) (SQK-LSK109)) were added following kit instructions. Sequencing was performed on MinION device using R9.4.1 flow cell to generate 186, 38153, and 31152 raw reads for the *E.coli* strains IPR_LC17, IPR_LC19 and IPR_LC20, respectively. Basecalling and demultiplexing of raw reads were performed using Guppy v3.6.1 ( https://nanoporetech.com). Adaptors were trimmed using PORECHOP v0.2.4 ( Wick et al., 2017) and poor quality reads, less than 500 bp and with an average quality score of below 10 were removed using NANOFLIT v2.8.0 ( De Coster et al., 2018).

*E.coli* PR_LC17 genome was assembled using Canu v2.2 ( Koren et al., 2017) while *E.coli* (IPR_LC19) and E.coli (IPR_LC20) were assembled using Flye v2.9.1 ( Kolmogorov et al., 2019). The taxonomy of the organism was confirmed using public databases for molecular typing and microbial genome diversity (PubMLST) ( Jolley et al., 2018). Quality of the assembled genomes was assessed using QUAST v5.1.0 ( Gurevich et al., 2013) and annotated using NCBI’s PGAP ( Tatusova et al., 2016). The assembly metrics and annotation summaries are as shown in Table 2.

**Table 2.** PGAP genome annotation of E.coli (IPR_LC17, IPR_LC19 & IPR_LC20 strains).

Feature	*E.coli* (IPR_LC17)	*E.coli* (IPR_LC19)	*E.coli* (IPR_LC20)
Genome size (Mbp)	5.08	4.70	5.66
GC content (%)	50.76	51.00	50.52
Sequencing coverage (X)	33	18	10
No. of contigs	2	2	13
N _50_ (bp)	4,984,001	4,606,403	4, 015,521
Genes (total)	5398	4849	6491
CDSs (total)	5277	4730	6367
Genes (coding)	1409	1604	2115
rRNAs	22	22	22
tRNAs	89	85	92
ncRNAs	10	12	10
CRISPR sequences	0	0	1

### Ethical considerations

The project was approved by Institute of Primate Research (IPR) review committee (ISERC/10/2020).

## Data Availability

IPR_LC20 Genbank: Escherichia coli strain IPR_LC20, whole genome shotgun sequencing project, Accession number JAOZEZ000000000: https://www.ncbi.nlm.nih.gov/nuccore/JAOZEZ000000000.1/ Sequence Read Archive (SRA): Oxford Nanopore Reads of Escherichia coli in camel production systems in Northern Kenya, Accession number SRR21998714: https://www.ncbi.nlm.nih.gov/sra/SRR21998714 IPR_LC19 Genbank: Escherichia coli strain IPR_LC19, whole genome shotgun sequencing project, Accession number JAOZFA000000000: https://www.ncbi.nlm.nih.gov/nuccore/JAOZFA000000000.1/ Sequence Read Archive (SRA): Oxford Nanopore Reads of Escherichia coli in camel production systems in Northern Kenya, Accession number SRR21998715: https://www.ncbi.nlm.nih.gov/sra/SRR21998715 IPR_LC17 Genbank: Escherichia coli strain IPR_LC17, whole genome shotgun sequencing project, Accession number JAOZFB000000000: https://www.ncbi.nlm.nih.gov/nuccore/JAOZFB000000000.1/ Sequence Read Archive (SRA): Oxford Nanopore Reads of Escherichia coli in camel production systems in Northern Kenya, Accession number SRR21998716: https://www.ncbi.nlm.nih.gov/sra/SRR21998716
