# Supplementary material for: Draft genome sequences of three emerging beta-lactamase-producing Escherichia coli in the camel production system in Northern Kenya
Source: F1000Res. 2023 May 16;11:1413. Originally published 2022 Nov 30. [Version 2] doi: 10.12688/f1000research.127990.2 (PMC10466007; doi:10.12688/f1000research.127990.2)
Supplement: Peer review history

# Peer review: Reviewer response for version 2

**DOI:** 10.5256/f1000research.147722.r173957

**Published:** 2023-08-29

**Authors:** Meenakshi S. Iyer

**Affiliations:** 1 National Centre for Biological Sciences, Tata Institute of Fundamental Research, Bengaluru, India

I have gone through the revised version of the manuscript. Most of the suggested changes have been addressed. I approve this version of the manuscript.

Are the datasets clearly presented in a usable and accessible format, and the assembly and annotation available in an appropriate subject-specific repository?

Yes

Are sufficient details of the sequencing and extraction, software used, and materials provided to allow replication by others?

Yes

Are the rationale for sequencing the genome and the species significance clearly described?

Partly

Are the protocols appropriate and is the work technically sound?

Yes

Reviewer Expertise:

Genomics, Transcriptomics, Anti-microbial resistance, Protein sequence-structure-funcion relationships, Database and algorithm development, Big-data analysis

I confirm that I have read this submission and believe that I have an appropriate level of expertise to confirm that it is of an acceptable scientific standard.

# Peer review: Reviewer response for version 1

**DOI:** 10.5256/f1000research.140538.r162944

**Published:** 2023-03-02

**Authors:** Janet Midega

**Affiliations:** 1 KEMRI-Wellcome Trust Research Programme, Kilifi, Kenya

The authors provide details of a study in which beta lactamase producing Ecoli from fecal samples are sequenced from Camels in Northern Kenya. Whilst this is an important study given the close interactions between human and camels in this part of Kenya, hence the likelihood of Animal to human transmission of beta lactamase producing E coli, It might help if the authors can write down a justification for why the study was conducted.

 Why was there a focus on beta-lactamase producing E coli only? For example is it the most important isolate in this (human and camel) population? 

 In total how many camels are represented in the study or were these random fecal samples? 

 Is it possible to be more precise about the location, for example provide GPS co-ordinates of the sampling locations - A county is a very wide area.

Are the datasets clearly presented in a usable and accessible format, and the assembly and annotation available in an appropriate subject-specific repository?

Yes

Are sufficient details of the sequencing and extraction, software used, and materials provided to allow replication by others?

Yes

Are the rationale for sequencing the genome and the species significance clearly described?

Partly

Are the protocols appropriate and is the work technically sound?

Yes

Reviewer Expertise:

Genomic epidemiology, Microbiology

I confirm that I have read this submission and believe that I have an appropriate level of expertise to confirm that it is of an acceptable scientific standard.

# Peer review: Reviewer response for version 1

**DOI:** 10.5256/f1000research.140538.r157281

**Published:** 2023-01-04

**Authors:** Meenakshi S. Iyer

**Affiliations:** 1 National Centre for Biological Sciences, Tata Institute of Fundamental Research, Bengaluru, India

In this manuscript the authors present a genome sequences of three *E. coli* strains isolated from camels in Northern Kenya. The importance of studying drug-resistant bacteria from livestock is obvious and these sequences are of course a very useful resource for the community. However I have a request for minor revisions. I found that the paper needs to address a few more points:

1. The authors need to provide more background on the need for sequencing *E. coli* samples from camel populations. There are some recent studies on beta-lactamase producing isolates from camle and other populations from Kenya. These studies (and more) can be referenced: Karegi I et al., 2022 DOI: https://doi.org/10.1016/j.ijid.2021.12.018; Anyanwu MU et al., 2012 https://doi.org/10.1155/2021/6630379; and Nueesch-Inderbinen M et al., 2020 https://doi.org/10.1016/j.sciaf.2020.e00274.
2. The results can be recorded in a separate section. Table 2 on genome annotation statistics can be moved to this section.
3. The results for the antibiotic susceptibility testing for the three isolates can be provided as a separate table.
4. The results of the PCR reactions for the 3 isolates describing which plasmids were detected from the samples need to be provided. These results can be corroborated with existing literature on the beta-lactamase encoding plasmid prevalence in Africa.
5. In Table 2, the statistics for partial genes detected in each strain can be listed. Overall, the authors can comment on the draft nature of the genome in the results section (% completeness, number of contigs etc.).

Are the datasets clearly presented in a usable and accessible format, and the assembly and annotation available in an appropriate subject-specific repository?

Yes

Are sufficient details of the sequencing and extraction, software used, and materials provided to allow replication by others?

Yes

Are the rationale for sequencing the genome and the species significance clearly described?

Partly

Are the protocols appropriate and is the work technically sound?

Yes

Reviewer Expertise:

Genomics, Transcriptomics, Anti-microbial resistance, Protein sequence-structure-funcion relationships, Database and algorithm development, Big-data analysis

I confirm that I have read this submission and believe that I have an appropriate level of expertise to confirm that it is of an acceptable scientific standard, however I have significant reservations, as outlined above.

## References

[1] : Antimicrobial Resistance Profile and Characterization of ESBLs in Escherichia coli from Camels in Northern Kenya. *International Journal of Infectious Diseases* .2022;116: 10.1016/j.ijid.2021.12.018 S7-S8 10.1016/j.ijid.2021.12.018 34954309

[2] : Prevalence and Traits of Mobile Colistin Resistance Gene Harbouring Isolates from Different Ecosystems in Africa. *Biomed Res Int* .2021;2021: 10.1155/2021/6630379 6630379 10.1155/2021/6630379 33553426PMC7847340

[3] : Antimicrobial resistant and extended-spectrum ß-lactamase (ESBL) producing Escherichia coli isolated from fecal samples of African dromedary camels. *Sci Afr* .2020;7: 10.1016/j.sciaf.2020.e00274 e00274 10.1016/j.sciaf.2020.e00274 34171008PMC7148658
